# New evidence of baobab (*Adansonia digitata*) pounding by western chimpanzees (*Pan troglodytes verus*)

**DOI:** 10.1007/s10329-026-01264-1

**Published:** 2026-05-27

**Authors:** Carlota F. Galán-Plana, Andreu Sánchez-Megías, Justinn Renelies-Hamilton, Manuel Llana, Samba Diallo, Amanda Barciela, Laia Dotras, Jordi Galbany, R. Adriana Hernandez-Aguilar

**Affiliations:** 1 Jane Goodall Institute Spain in Senegal, Dindefelo Biological Station, Dindefelo, Senegal; 2 Department of Primate Behavior and Evolution, Max Planck Institute for Evolutionary Anthropology, Leipzig, Germany; 3 Department of Social Psychology and Quantitative Psychology, Faculty of Psychology, University of Barcelona, Barcelona, Spain; 4 Department of Clinical Psychology and Psychobiology, Faculty of Psychology, University of Barcelona, Barcelona, Spain; 5 Institute of Neurosciences, University of Barcelona, Barcelona, Spain; 6 Serra Hunter Programme, Generalitat de Catalunya, Barcelona, Spain

**Keywords:** Baobab cracking, Extractive foraging, Proto-tool use, Anvil use, Passive hammer use

## Abstract

Percussive technology in wild chimpanzees (*Pan troglodytes*) shows significant behavioral variation across Africa. An example is baobab (*Adansonia digitata*) fruit pounding, which is only known to occur in the Bandafassi Arrondissement in the Kedougou region (Senegal) and in the Bafing area (Mali). The extent to which this proto-tool use behavior varies between populations is poorly understood. Here, we present a preliminary report of the baobab-pounding behavior of savanna chimpanzees from Dindefelo, Senegal. During the peak baobab fruiting season from November 2021 to January 2022, we obtained evidence of baobab pounding, both primate archaeological evidence (fruit remnants smeared on anvils and cracked, empty baobab shells) and camera trap recordings. We also studied chimpanzee baobab consumption using macroscopical fecal analysis. In addition, in the peak baobab fruiting season from 2019 to 2020, we opportunistically obtained hand-held camera videos of baobab pounding. Dindefelo chimpanzees opened baobab using two pounding and one non-percussive techniques. We recorded all age and sex classes performing baobab pounding, although only adolescents and adults succeeded in opening the fruit. Baobab pounding occurred on the ground (against rocks and baobab roots) and on the trees (against baobab horizontal branches). The apes held the fruit by the stem or the pericarp while cracking it against an anvil. In addition, the fecal analysis data suggest that baobab seed reingestion is common at Dindefelo. These findings expand our knowledge on the diversity of chimpanzee baobab-pounding behavior.

**Supplementary Information:**

The online version contains supplementary material available at 10.1007/s10329-026-01264-1.

## Introduction

Percussive technology is the application of forceful strikes of a solid body against another to achieve a goal (sensu Marchant and McGrew 2005). Only a few species of non-human primates (hereafter primates) use this technology for extractive foraging, to access resources that are difficult to obtain. One type of percussive technology involves the combined use of a hammer and an anvil (Boesch and Boesch 1981), which is customary only in a few communities of West African chimpanzees (*Pan troglodytes verus*) (Boesch and Boesch 1983; McGrew 1992; Whiten et al. 2001), among robust capuchin monkeys from Brazil (*Sapajus* spp.) (Falótico and Ottoni 2016; Falótico et al. 2018; Falótico 2022; Fragaszy et al. 2004), and Burmese long-tailed macaques (*Macaca fascicularis aurea*) (Falótico et al. 2017; Luncz et al. 2017; Malaivijitnond et al. 2007). Hammer-and-anvil use is habitual in one community of white-faced capuchin monkeys (*Cebus capucinus*) (Barrett et al. 2018).

On some occasions, animals use a hammer to hit an object without using an anvil (Shumaker et al. 2011). Hammering is known in wasps, birds, and mammals (reviewed in Shumaker et al. 2011). Among primates, capuchin monkeys use hammering to crack open dried-out branches, seeds, and oysters, break tubers into smaller pieces, process cacti, and access vertebrate burrows (reviewed in Shumaker et al. 2011). When tools were unavailable, they hammered oysters with pieces of other oysters (Fernandes 1991). There is also a report of a capuchin hitting nuts against other nuts without success (Struhsaker and Leland 1977). Among chimpanzees, a hammering behavior found in Bossou (Guinea) is pestle-pounding, by which the apes use the leaf-petiole of oil-palm trees (*Elaeis guineensis*) to pound the palm crown and access the edible products in its heart (Yamakoshi and Sugiyama 1995; reviewed in Shumaker et al. 2011).

Another type of percussive technology is food pounding (Van Lawick-Goodall 1968; Whiten et al. 2001), also called anvil use (McGrew et al. 1999) or passive hammer use (Harmand and Arroyo 2023). This behavior entails cracking embedded foods against substrates (e.g., branches, trunks, roots, stones, or the ground) to access their interior (Van Lawick-Goodall 1968; McGrew et al. 2003; Whiten et al. 2001). It is considered a proto-tool use since the anvil is never directly manipulated, although it functions in a similar way as a tool (Shumaker et al. 2011). Food pounding occurs in fishes, birds, and mammals (reviewed in Shumaker et al. 2011). Among primates, it is most widespread among chimpanzees, although it occurs in other species, namely Nicobar long-tailed macaques (*M. f. umbrosus*) to open coconuts (*Cocos nucifera*) (Mazumder and Kaburu 2020; Pal et al. 2018).

Chimpanzee fruit pounding is widespread across Africa. Western chimpanzees (*P. t. verus*) pound the fruits of *Adansonia digitata* (Gašperšič & Pruetz 2008; McGrew et al. 2003; Marchant and McGrew 2005; Duvall 2008; Pruetz et al. 2020), *Afraegle paniculata* (Lapuente 2020), *Saba senegalensis* (Pruetz et al. 2020), *Strychnos* spp. (Boesch and Boesch 1983; Matsuzawa and Yamakoshi 1996; Pruetz et al. 2020; Whiten et al. 2001), and *Treculia africana* (Koops et al. 2010; Whiten et al. 2001). In Seringbara (Guinea), the big *T. africana* fruits are presumably pounded to fracture them into smaller pieces, so this is not considered extractive foraging (Koops et al. 2010). On the other hand, Eastern chimpanzees (*P. t. schweinfurthii*) also pound *Conopharyngea* (Whiten et al. 2001), *Strychnos* (Goodall 1986; Hicks et al. 2019; McGrew et al. 1999) and *Balsamocitrus* (Whiten et al. 2001). Lastly, Nigeria-Cameroon chimpanzees (*P. t. ellioti*) pound the fruits of *Symphonia globulifera* (Dutton and Chapman 2015). Besides fruits, Western and Eastern chimpanzees pound termite (Isoptera) mounds (Luncz and Boesch 2015; Hicks et al. 2019), Eastern chimpanzees pound African giant snails (*Achatina* spp.) (Hicks et al. 2019), and Central chimpanzees (*P. t. troglodytes*) pound hinge-back tortoises (*Kinixys* spp.) (Hicks et al. 2019; Pika et al. 2019). Chimpanzee pounding behaviors are reviewed in Harmand and Arroyo (2023).

Among chimpanzees, cultural variations between communities exist for hammer-and-anvil use (Humle and Matsuzawa 2004; Luncz et al. 2012; Luncz and Boesch 2015; Whiten et al. 2001), but these remain unstudied for anvil use. A rarely studied anvil use behavior in chimpanzees is baobab (*A*. *digitata*) pounding to crack open the hard-shelled fruits of this species. This behavior is restricted to West African savannas, where chimpanzees coexist with baobabs (McGrew et al. 2003). It was first reported for unhabituated chimpanzees in Assirik (Senegal) using primate archeological data and direct observations (Baldwin 1979; Marchant and McGrew 2005; McGrew et al. 1988, 2003). Later, the first study of habituated savanna chimpanzees, in Fongoli (Senegal), provided the first long-term behavioral data (Cissé and Pruetz 2009; Pruetz et al. 2020; Lindshield et al. 2021a). This behavior has also been reported to occur at Bandafassi and Mt. Bagnomba in Senegal (Gašperšič & Pruetz 2008). Additionally, there is acoustic and video evidence that the chimpanzees in the Bafing area in Mali also crack baobab (Duvall 2008; M Aranjelovic, scientist, personal communication). Given that behavioral data are only available for Fongoli, we have a restricted knowledge on how this proto-tool use varies among chimpanzee communities.

Baobab fruits are an important nutritional resource for the chimpanzees (Lindshield et al. 2021a). In Fongoli, the apes reingest baobab seeds from their feces to extract the kernels’ protein and fat, since the seed coat softens during the first digestion (Lindshield et al. 2021a). Baobab is also a key dietary and medicinal food across African human communities (Buchmann et al. 2010).

**Fig. 1:**
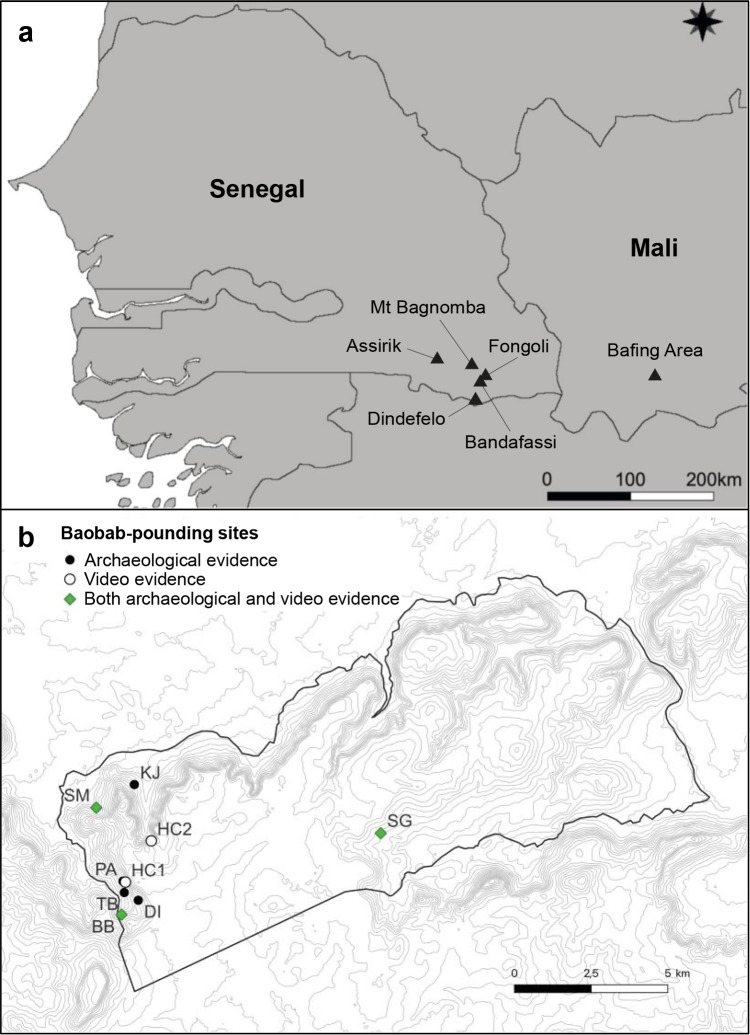
Location of **a** the six chimpanzee study sites in West Africa where baobab pounding has been reported, including Dindefelo, and **b** the baobab-pounding sites within Dindefelo where we obtained archeological and/or video evidence of this behavior. For one site where we obtained a hand-held camera video, we could not record GPS coordinates

Here, we present the first evidence of baobab pounding among chimpanzees from Dindefelo (Senegal), increasing to six the locations where this percussive technology has been reported (Fig. 1a). We recorded baobab-pounding behavior using camera traps, examined anvils using primate archeological techniques, and studied chimpanzee baobab consumption frequency using macroscopical fecal analysis. We provide the first comparison of this percussive behavior between chimpanzee communities, increasing our knowledge on savanna chimpanzee proto-tool use and material culture.

## Materials and methods

### Study site and subjects

We studied wild, non-habituated, non-provisioned chimpanzees at the Dindefelo Community Nature Reserve (hereafter Dindefelo), in the Kedougou region, southeastern Senegal (Fig. 1b). Dindefelo occupies 140 km^2^ alongside the border with Guinea, 40 km southeast of the Niokolo-Koba National Park (Senegal). Dindefelo encompasses multiple vegetation types: grassland, shrubland, wooded grassland, woodland, gallery forest, forest, dense bamboo (*Oxytenanthera abyssinica*) woodland, and cropland. There is an extensive dry season (November–May) and a rainy season (June-October). Mean annual temperature is 28.5ºC and mean annual precipitation is 1129 mm. We have identified 53 adult chimpanzees in Dindefelo. The reserve and its surrounding area have a total of 14 villages and hamlets. For further description of the study site see Dotras et al. (2024) and Sánchez-Megías et al. (2024).

### Data collection

During the baobab fruiting season from November 2021 to January 2022, we deployed camera traps (Bresser DL-30MP, Browning BTC-T-4 K Edge, and Bushnell Trophy Cam HD Essential E3) on five locations, and we recorded baobab pounding in three of them (Fig. 1b). We selected these locations based on previous behavioral, archeological and acoustic evidence of baobab pounding, and fruit abundance. We placed camera traps to record both baobab trunks and potential anvils around them. Camera traps recorded upon detecting movement for 1 min of footage with a 1-s delay between consecutive videos. During this study, we found and studied two more baobab-pounding sites where no cameras were placed. In addition, during the baobab fruiting season of 2019-2020, we opportunistically recorded from afar three videos of baobab pounding with a hand-held camera (Sony DSC-H400; Fig. 1b).

### Data analysis

**Fig. 2:**
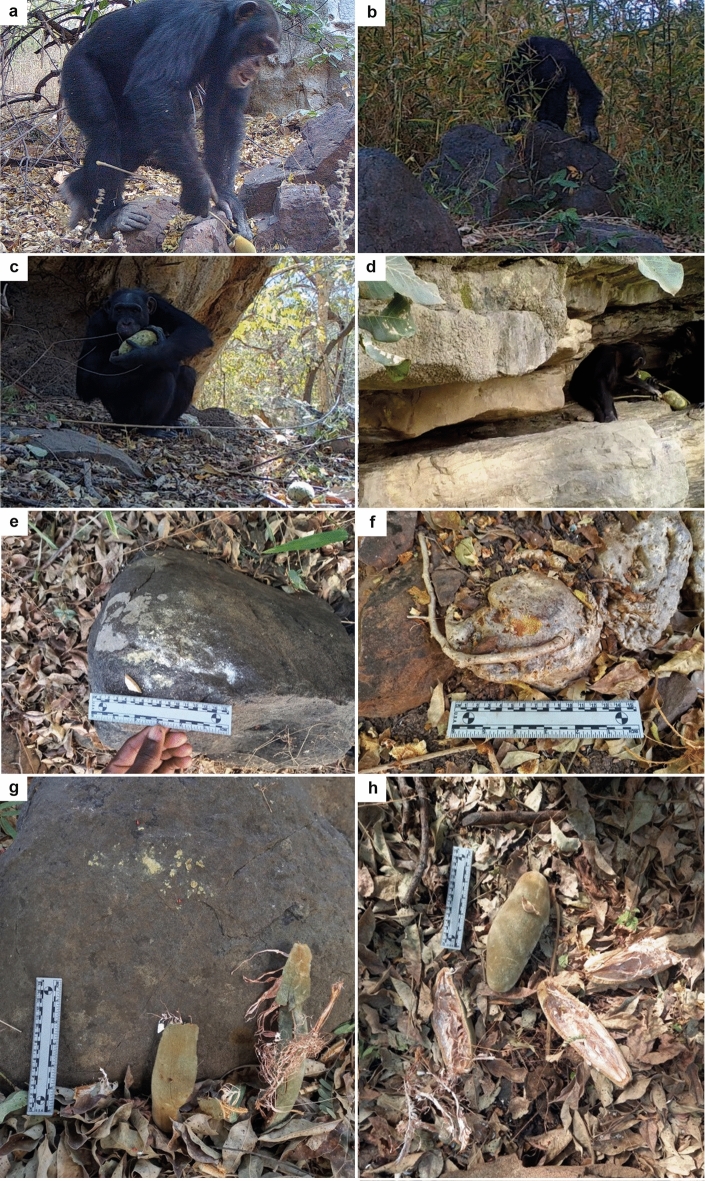
**a** Male adult chimpanzee pounding a baobab fruit against a boulder while holding it by the stem, **b** male adult chimpanzee pounding a baobab fruit against a boulder while holding it by the pericarp, **c** female adult chimpanzee trying to open a baobab fruit by biting it, **d** infant chimpanzee pounding a baobab fruit against a rocky outcrop, **e** baobab fruit remnants smeared against the surface of a boulder, **f** baobab fruit remnants smeared against the surface of a baobab tree root, **g** baobab fruit remnants smeared against the surface of a boulder and cracked, empty baobab fruit shells found next to the anvil, **h** cracked, empty baobab fruit shells found next to a boulder

We analyzed the videos in which chimpanzees performed baobab pounding. We defined a baobab-pounding event as the consecutive footage of the same individual using the same anvil to open the same baobab fruit, without leaving the camera frame or stopping the pounding for more than 15 min (following Boesch et al. 2017). We obtained the GPS location of each baobab-pounding site using a Garmin Etrex 32x. For each event, we noted the date, time, duration (s), hand used to pound the fruit, body position, anvil material, number of hits against the anvil, whether the individual managed to open the fruit, and fruit-grasping technique (Fig. 2a, b; Table 1). We recorded individual sex and age (Table 1). We did not identify the sex of infants.

**Table 1:** Variables studied for each baobab-pounding event

Variable	Description
Date	Day, month and year in which the event occurred
Time	Hour of the day in which the event occurred
Site	Location in which the event occurred
Sex	Whether the chimpanzee was male, female, or unknown
Age	Whether the chimpanzee was an infant, a juvenile, an adolescent, or an adult
Event duration	Number of seconds that the event lasted
Hand used	Whether the chimpanzee held the fruit with the right and/or the left hand
Body position	Whether the chimpanzee was standing bipedally, standing tripedally, or sitting down while pounding
Anvil material	Whether it was a stone or wooden anvil
Hits against the anvil	Number of times that the chimpanzee pounded the fruit
Grasping technique	Whether the chimpanzees held the fruit by the stem and/or the pericarp while pounding it
Cracking success	Whether the chimpanzee succeeded in opening the fruit
Pounding rate	Number of times that the chimpanzee pounded the fruit per second

In 2021 and 2022, we studied seven baobab-pounding sites and placed camera traps in five sites. We defined a baobab-pounding site as the location containing one baobab tree, one or more associated anvils, and evidence that chimpanzees had cracked baobab there. In these sites, we collected indirect baobab-pounding evidence using primate archeology techniques, following Marchant and McGrew (2005). We examined potential anvils at these sites, and we identified those that presented macroscopic remnants of baobab fruit smeared on their surface as anvils (Fig. 2e–g). Evidence of pounding also included cracked, empty baobab shells (Fig. 2g, h). We recorded anvil size in the three perpendicular axes (at their widest point, cm), whether anvils were fixed or movable, whether their material was stone or wood, and the distances from each anvil to the associated baobab tree and between anvils (cm).

**Fig. 3:**
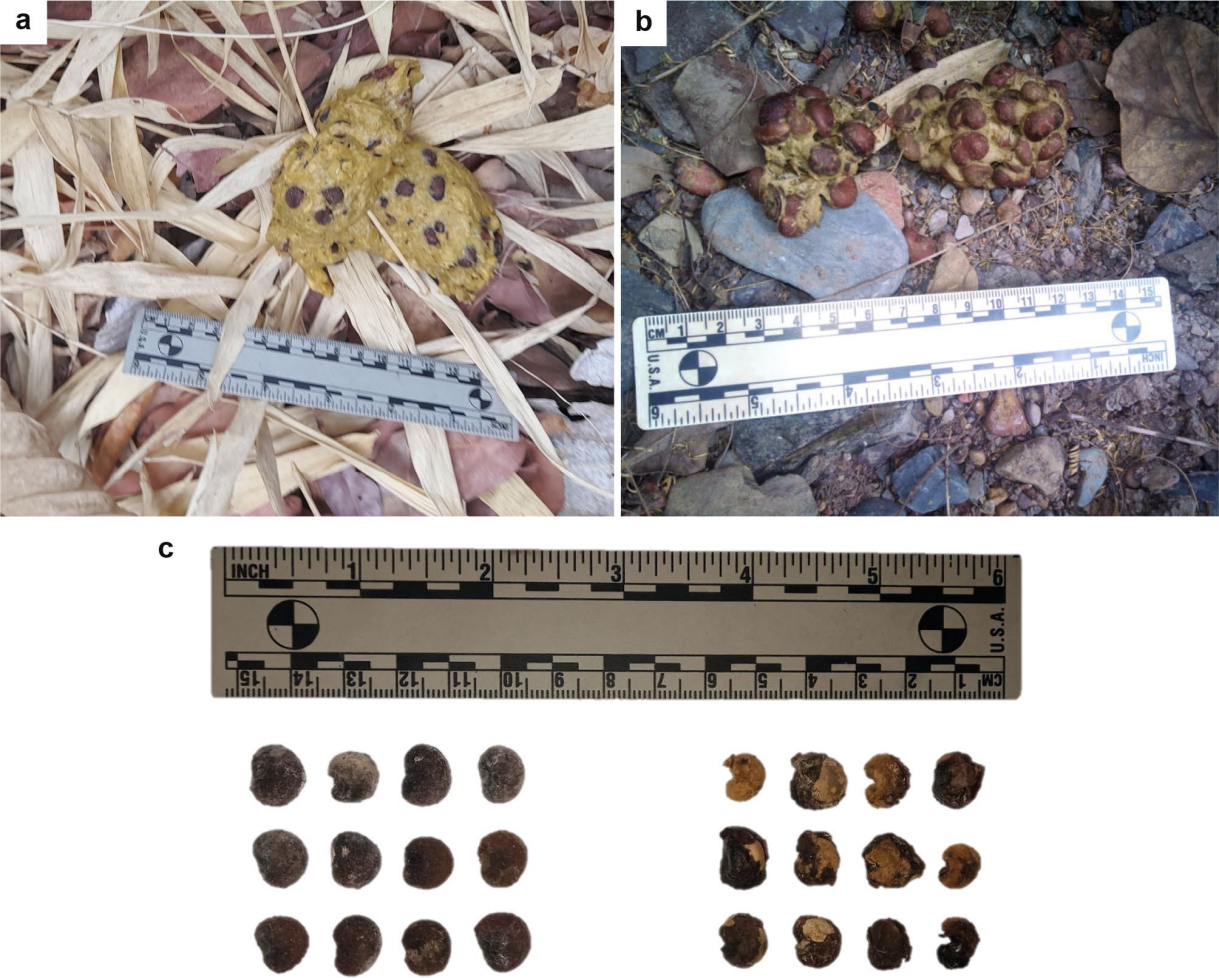
**a**, **b** Fresh chimpanzee feces containing baobab seeds, **c** baobab seeds ingested a single time (right) and reingested baobab seeds (left)

To assess baobab consumption frequency, we macroscopically analyzed 53 (< 24 h) chimpanzee fecal samples collected under fresh nests to reduce pseudo-replication (Fig. 3a, b). We counted the total number of baobab seeds and the number of reingested seeds (Fig. 3c) in each fecal sample (following Lindshield et al. 2021a). We calculated baobab seed density dividing the number of seeds by the fecal mass (g), and the proportion of reingested seeds per fecal sample. We obtained mean baobab seed weight (g) after averaging the weight of 50 baobab seeds found in feces. With this, we calculated the proportion of fecal weight corresponding to baobab seeds. We also recorded the presence of baobab seeds in fresh feces found opportunistically during daily fieldwork from 2014 to 2021.

## Results

We found a total of ten baobab-pounding sites in Dindefelo. Between 2021 and 2022, we obtained 3195 video recordings, 149 of which contained chimpanzees, and nine of which included chimpanzees pounding baobab. We obtained video recordings of chimpanzees performing baobab pounding in three of the five camera trap locations. These videos, alongside the three hand-held camera videos obtained between 2019 and 2020, totaled six locations where baobab-pounding behavior was video recorded in Dindefelo (Fig. 1b). For the remaining four baobab-pounding sites, we only obtained primate archaeological evidence of this behavior.

We recorded seven baobab-pounding events performed by both sexes and all age classes (Table 2). Infants and juveniles pounded for longer (*n* = 3, Mean ± SD: 89 ± 37.99 s, range = 60—132) than adolescent and adults (*n* = 4, 42.5 ± 17.29 s, range = 20–60). One infant and one juvenile did not succeed in opening the fruit (Table 2). In three cases, the recording stopped before the event had concluded, so we could not note whether these events were successful. Baobab-pounding events lasted for 62 ± 35 s (range = 20–132) and their duration decreased with age (Table 2). An adult displayed the shortest successful event to crack open one fruit in 20 s (Table 2). Chimpanzees pounded fruits a mean of 10.4 ± 7.1 times (range = 2–22), at an average rate of 1.15 ± 0.4 hits per s (range = 0.6–1.8) (Table 2).

**Table 2:** Characteristics of the baobab-pounding events recorded

Sex	Age	Event duration (s)	Hand used	Body position	Hits against the anvil	Grasping technique	Cracking success	Pounding rate (hits/s)
-	I	132	Both	Shifting (bipedal and tripedal)	22	Stem	No	0.6
F	J	75	Both	Shifting (bipedal and sitting)	12	Pericarp and stem	No	0.7
-	J	60	Right	Sitting (while holding onto a branch)	9	Pericarp	-	1.8
M	Ad	60	Left	Sitting	6	Pericarp	-	1.5
-	Ad	51	Left	Tripedal	5	Stem	Yes	1
M	A	39	Left	Bipedal	17	Pericarp	-	1.5
M	A	20	Right	Tripedal	2	Stem	Yes	1

Age classes are infant (I), juvenile (J), adolescent (Ad), and adult (A). Sex classes are male (M) and female (F). Dashes (-) indicate information not available

Dindefelo chimpanzees opened the fruits using three techniques. Two were proto-tool use techniques: a chimpanzee pounded the fruit against an anvil, either holding the fruit by the stem (*n* = 4; Table 2; Fig. 2a) or by the pericarp (*n* = 4; Table 2; Fig. 2b). In one event, a juvenile held the fruit by the pericarp and later by the stem (Table 2). The third technique was non-percussive: a chimpanzee bit the fruit directly without pounding it (*n* = 1; Fig. 2c). In the only non-percussive event we recorded, the chimpanzee was not successful in opening the fruit.

In five events, the hand used to grasp the fruit and the body position adopted while pounding did not change within the event. While grasping a fruit with one hand, they used their lips, tongue, or the other hand to extract and eat the fruit pulp and seeds (Supplementary Video 4). The adults and adolescents we observed used the same hand for holding, in most cases the left hand, whereas a juvenile and an infant switched hands within the event (Table 2). Chimpanzees adopted three different body positions when pounding and sometimes shifted between two body positions (Table 2); bipedal and tripedal stances were the most common. Adults completed pounding events without switching positions, whereas one infant and one juvenile switched positions before completing the pounding event.

The apes either cracked baobab fruits on tree branches (Supplementary Video 1) or descended to the ground to do so against stone anvils (Fig. 3a, b, e, g; Supplementary Videos 2, 3). Indirect pounding evidence was also found on a root anvil (Fig. 2f). No evidence of cracking directly on hard ground or tree trunks was found. The average distance between a stone anvil and its nearest baobab tree (stone-to-trunk distance, sensu Marchant and McGrew 2005) was 4 ± 2.6 m (*n* = 14, range = 0.5–7.3). All stone anvils were fixed*.* Anvils measured on average 109.6 ± 73.1 cm in length (*n* = 14, range = 37.9–252), 66.7 ± 28.5 cm in breadth (*n* = 14, range = 18.2–140), and 55.7 ± 37.2 cm in height (*n* = 14, range = 24.4–154.2) (Table 2). We recorded one pounding event on a large, stratified rock outcrop (Fig. 2d). The chimpanzee pounded the fruit against a horizontal surface of the outcrop (Fig. 2d). Additionally, we recorded chimpanzees transporting one or more baobab fruits both on the ground and on tree branches. Chimpanzees left cracked, empty shells on the ground close to anvils or tree trunks (Fig. 2g,h).

**Fig. 4:**
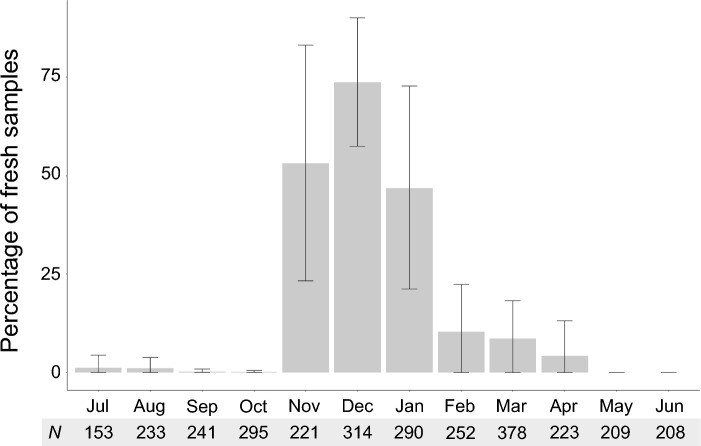
Total number of chimpanzee fresh feces collected each month from 2014 to 2021, and the percentage that contained baobab seeds, which were identified macroscopically in the field

Between 2014 and 2021, chimpanzee baobab consumption peaked from November to January (Fig. 4). We found baobab seeds in 29 out of the 53 feces (54.7%), which represented 73.7% of the feces collected in December and 76.5% of the feces collected in January (Fig. 3a, b). Baobab seeds weighted 0.393 g on average. Baobab seed density averaged 115.9 ± 68.1 seeds per g of fecal mass (range = 4.6–248.5) representing 43.2 ± 25.4% of total fecal mass (range = 1.7–92.7). Reingested seeds occurred in 89.7% of feces containing baobab seeds, representing 27.7 ± 22.3% of the total number of baobab seeds (range = 0–100; Fig. 3c).

## Discussion

We provide the first evidence that wild savanna chimpanzees from Dindefelo use anvils to crack open baobab fruits, increasing to six the chimpanzee study sites where this percussive technology is known to occur (Fig. 1a). Although Dindefelo chimpanzees use tools for extractive foraging (A.P.E.S. Wiki and Dindefelo Team 2025; Boesch et al. 2020; Kühl et al. 2019; Sánchez-Megías et al. 2024), baobab pounding is the first percussive technology observed in this population. Despite the presence of oil-palm (*Elaeis guineensis*) nuts, *Saba senegalensis* and *Strychnos* fruits in Dindefelo, there is no evidence to date that Dindefelo chimpanzees pound them, contrary to what has been reported for conspecifics in other West African study sites (Bossou, Guinea: Humle and Matsuzawa 2004; Fongoli, Senegal: Pruetz et al. 2020; Sapo Forest, Liberia: Anderson et al. 1983; Taï, Ivory Coast: Boesch and Boesch 1990; Tiwai Island, Sierra Leone: Whitesides 1985; Yealé, Ivory Coast: Humle and Matsuzawa 2004; see review by Harmand and Arroyo 2023).

Here we compare our preliminary data from Dindefelo with the evidence from the five other study sites where baobab pounding is known to occur (Fig. 1a). Of these, Fongoli is the only site where chimpanzees are habituated to human observers (Lindshield et al. 2021b). We used camera trapping for the first time to study the baobab-pounding behavior of non-habituated savanna chimpanzees. Dindefelo chimpanzees displayed two techniques in the context of baobab-fruit consumption. They opened the fruit by cracking it against an anvil, which concurs with what has been reported for the other study sites (Table 3). We observed one individual trying to open the fruit with the teeth (Fig. 2c) without success, and thus it is possible that Dindefelo chimpanzees open baobab fruit with their teeth. In Fongoli, the apes opened 45% of the baobab fruits they ate with their teeth alone, and the rest involved pounding (Pruetz et al. 2020). In Dindefelo, the apes hit baobab a mean of ten times before cracking it, similarly to what has been reported in Assirik (mean = 16 hits, range = 3–56, N = 22; Baldwin 1979) and Fongoli (median = 13 hits, range = 2–172, sample size not reported; Cissé and Pruetz 2009). In Dindefelo, the chimpanzees pounded baobab at a similar rate to what has been reported in Assirik (mean ≈ 1 hit/s; Baldwin 1979).

**Table 3:** Anvils used to pound baobab fruits at the six sites where baobab pounding is known to occur

Site	Tree branch	Tree trunk	Tree root	Boulder	Outcrop	Hard earth	References
Assirik, Senegal	Yes	Yes	Yes	Yes	-	-	(Baldwin 1979; McGrew et al. 2003; Marchant and McGrew 2005)
Fongoli, Senegal	Yes	Yes	Yes	Yes	Yes	Yes	(Cissé and Pruetz 2009; Pruetz et al. 2020)
Bandafassi, Senegal	Yes	-	Yes	Yes	-	Yes^1^	(Gašperšič and Pruetz 2008)
Mt. Bagnomba, Senegal	Yes	-	Yes	Yes	-	Yes^1^	(Gašperšič and Pruetz 2008)
Dindefelo, Senegal	Yes	-	Yes	Yes	Yes	-	This study
Bafing area, Mali	-	-	?	?	-	-	(Arandjelovic et al. 2016)

Interrogation marks (?) indicate that in the only baobab-pounding video available for the Bafing area (Mali), the anvil appears to be either a tree root or a boulder. Dashes (-) indicate there is no evidence confirming the use of this type of anvil

^1^The ‘mountainous’ sites reported in Gašperšič and Pruetz (2008) most likely refer to Bandafassi and Mt. Bagnomba, but this is not explicitly stated

While pounding, Dindefelo chimpanzees held the fruits by the stem or by the pericarp, same as has been reported for Fongoli chimpanzees (Cissé and Pruetz 2009). In the latter, holding the stem was more frequent than holding the pericarp (Cissé and Pruetz 2009). Preference for fruit grasping technique remains unknown in Dindefelo. Assirik chimpanzees were only observed holding the fruit by the stem for cracking (Baldwin 1979), but direct observations were infrequent at this study site. In the only video available for the Bafing area, a chimpanzee held the fruit by the pericarp (Arandjelovic et al. 2016). In Dindefelo, adolescents and adults used one hand consistently during a pounding event, while an infant and a juvenile switched hands. Hand preference in chimpanzee percussive technology has been described at two nut-cracking sites, Taï and Bossou, where most chimpanzees used the same hand consistently for this tool-use activity (reviewed in McGrew et al. 1999). In Dindefelo, most of the positions that the chimpanzees assumed while pounding (bipedal, tripedal, sitting and shifting; Table 2) have also been reported for Fongoli (Cissé and Pruetz 2009). In addition, we also observed a chimpanzee pounding while sitting on a big horizontal branch and holding onto a smaller branch with one hand.

Our preliminary data suggest that older individuals are more proficient in cracking baobab. Tool-use behaviors are acquired during ontogenetic development. An example among chimpanzees is nut-cracking, which is known to be socially learned and culturally transmitted (Boesch and Boesch 1993; Biro et al. 2006; Humle and Matsuzawa 2004; Luncz et al. 2012; Whiten et al. 2009). Similarly, proto-tool use behaviors appear to be acquired during an animal’s development (Jacobs and Osvath 2016). In our study, the lower rates of baobab-cracking success and the higher pounding rates exhibited by youngsters are similar to what has been reported from Fongoli (Cissé and Pruetz 2009), which suggest that adults are more skilled in opening baobab. Switching hands during tool use may be part of the process of individual learning (Biro et al. 2006). The fact that we only observed young chimpanzees switching hands while pounding, supports the idea that hand-switching may also play a part in the acquisition of proto-tool use behaviors.

In this study, we report measurements of anvils used by Dindefelo chimpanzees to pound baobab fruits. We could not find any published data on the measurements of anvils used by chimpanzees elsewhere. The anvils used by Dindefelo chimpanzees were both terrestrial (boulders, an outcrop and an exposed root) and arboreal (horizontal branches), and were all non-movable. We did not find evidence that they pound baobab fruits against trunks, hard earth, or movable anvils. Fongoli chimpanzees frequently pounded baobab fruits against tree branches, less frequently against stones, and rarely against tree trunks or hard earth (Cissé and Pruetz 2009). Assirik chimpanzees were observed to use tree branches and trunks as anvils (Baldwin 1979) and indirect evidence of pounding against terrestrial non-movable anvils was found (Marchant and McGrew 2005; McGrew et al. 2003). At Bandafassi and Mt. Bagnomba, evidence of pounding against tree branches and roots, stones, and hard earth was found (Gašperšič and Pruetz 2008). In the only video available for Bafing, an individual uses a terrestrial anvil, but it is unclear whether it is a movable or fixed boulder, or an exposed root (Arandjelovic et al. 2016). Given that only few instances have been observed in Assirik, Dindefelo, and Bafing, anvil type preference remains unknown for these sites. Fongoli chimpanzees transported baobab fruits, as many as five at a time, up to 1 km (Cissé and Pruetz 2009). We recorded Dindefelo chimpanzees transporting baobab fruits on horizontal tree branches and on the ground, but total transport distances remain unknown.

We have acoustic and primate archaeological evidence suggesting that the baobab-pounding sites reported in this study had been used by the apes in previous years [Dindefelo field assistants, personal communication; Jane Goodall Institute Spain (JGIS), unpublished data]. However, we do not know whether Dindefelo chimpanzees reuse the same anvils. Anvil reuse is known for other percussive behaviors, such as nut-cracking at Bossou (Carvalho et al. 2009). Chimpanzees at Dindefelo and the other Senegalese sites use both stone and wooden anvils, and arboreal and terrestrial techniques (Table 3). The video from the Bafing area shows a chimpanzee pounding against what seems to be a stone or a baobab exposed root (Table 3). Further behavioral and primate archaeology studies will allow comparisons between these sites in pounding technique and anvil type preference, baobab fruit transportation distances, and anvil reuse.

West African savanna chimpanzees face highly-seasonal environments marked by water scarcity, patchily-distributed food, and high temperatures (Lindshield et al. 2021b). Percussive technology may facilitate access to embedded, valuable resources in these harsh habitats (Lindshield et al. 2021a). An example is baobab fruit, which is an important dietary item for savanna chimpanzees in Senegal (Baldwin 1979; Bogart 2009; McGrew et al. 1988, 2003; Lindshield et al. 2017, 2021a; Pruetz 2006; Pruetz et al. 2020). In Dindefelo, baobab fruit availability peaked in October and November (JGIS, unpublished data). It is from November to January when chimpanzee baobab consumption was highest, as demonstrated by fecal analysis (Fig. 4). However, once ripe, the matrix of these fruits is dry and powdery, remaining edible all year round (Lindshield et al. 2017), allowing Dindefelo chimpanzees to eat them in smaller amounts outside of the peak fruiting season (Fig. 4), as occurs in Fongoli (Pruetz et al. 2020). Baobab pounding in the latter site was more frequent during the baobab consumption peak (Lindshield et al. 2017, 2021a; Pruetz et al. 2020), which spans the same months as in Dindefelo. In Assirik and Fongoli, chimpanzees reingest baobab seed kernels, which are rich in protein and fat (Baldwin 1979; Bertolani and Pruetz 2011; Lindshield et al. 2017, 2021a). Similarly, we found evidence that Dindefelo chimpanzees reingested seed kernels (Fig. 3c). At Fongoli, pounding unripe baobab fruits allows the apes to access this high-quality food which abounds during a period when other fruits present low availability (Pruetz et al. 2020; Lindshield et al. 2021a). Dindefelo had medium–low fruit availability during the baobab availability peak (JGIS, unpublished data). In savannas, tool use for extractive foraging exhibits seasonal patterns (Lindshield et al. 2021b). Proto-tool use in this habitat may also follow a similar pattern, as suggested by baobab-pounding evidence (direct observations from Fongoli: Pruetz et al. 2020; fecal analysis from Assirik: Baldwin 1979; and our fecal analysis data from Dindefelo). Although percussion is not always necessary to crack baobab fruits, it likely improves feeding efficiency by increasing opening and ingestion rates (Lindshield et al. 2021a; Pruetz et al. 2020). In sum, percussive technology to crack baobab fruits and seed reingestion may be important chimpanzee feeding strategies to improve nutrient intake rates in savannas. Baobab pounding supports the idea that technological behaviors are key for chimpanzees to survive in savanna environments (Pruetz et al. 2020).

Wild chimpanzee percussive technology shows cultural variation among populations (Marchant and McGrew 2005; McGrew 1992; Luncz et al. 2012; Whiten et al. 2001, 2009). The most studied example is nut-cracking using hammer and anvil in western chimpanzees (Boesch-Achermann and Boesch 1993; Biro et al. 2006; Humle and Matsuzawa 2004; Luncz et al. 2012; Whiten et al. 1999, 2009). Intraspecific variability in proto-tool use behaviors has also been reported (Whiten et al. 1999, 2009). We provide preliminary data from a new baobab-pounding study site, which allowed us to make comparisons with what has been reported for the four other sites in Senegal where this behavior has been reported. In addition, we include the first video evidence of this percussive behavior from the Bafing area (Arandjelovic et al. 2016). Studying behavioral differences between populations living under similar ecological constraints, such as in the forementioned baobab-pounding sites, is key to detect cultural differences. However, detailed data on baobab pounding are only available for Fongoli (Cissé and Pruetz 2009; Lindshield et al. 2021a; Pruetz et al. 2020). Camera traps are a promising tool to study this percussive behavior in the unhabituated chimpanzee populations that co-occur with baobab, as it is the case for other behaviors of unhabituated savanna chimpanzees (Boyer-Ontl and Pruetz 2014). Further studying baobab pounding will expand our knowledge on the diversity of technological behaviors used by savanna chimpanzees to exploit harsh environments, which has important implications for understanding the evolution of early hominin technology (Lindshield et al. 2021b).

## Supplementary Information

Below is the link to the electronic supplementary material.

Supplementary file1 Video S1. A juvenile chimpanzee pounds a baobab fruit against a tree branch while grasping the fruit by the pericarp (MP4 20604 KB)

Supplementary file2 Video S2. An adult male pounds a baobab fruit terrestrially against a rock anvil while grasping the fruit by the pericarp (MP4 13292 KB)

Supplementary file3 Video S3. An adult male pounds a baobab fruit against a rock anvil while holding it by the stem until the fruit cracks open. He then uses his lips to consume the interior (MP4 14091 KB)

Supplementary file4 Video S4. An adult male extracts the interior of a baobab fruit with his teeth and eats it while holding the fruit with his right hand (MP4 13192 KB)
